# Cytokine expression in mice exposed to diesel exhaust particles by inhalation. Role of tumor necrosis factor

**DOI:** 10.1186/1743-8977-3-4

**Published:** 2006-02-20

**Authors:** Anne T Saber, Nicklas R Jacobsen, Jette Bornholdt, Sanna L Kjær, Marianne Dybdahl, Lotte Risom, Steffen Loft, Ulla Vogel, Håkan Wallin

**Affiliations:** 1 National Institute of Occupational Health, Lersø Parkallé 105, 2100 Copenhagen, Denmark; 2 Institute of Public Health, Copenhagen University, Øster Farimagsgade 5, opg. B, 2.sal; postbox 2099, 1014 Copenhagen K, Denmark

## Abstract

**Background:**

Particulate air pollution has been associated with lung and cardiovascular disease, for which lung inflammation may be a driving mechanism. The pro-inflammatory cytokine, tumor necrosis factor (TNF) has been suggested to have a key-role in particle-induced inflammation.

We studied the time course of gene expression of inflammatory markers in the lungs of wild type mice and *Tnf-/- *mice after exposure to diesel exhaust particles (DEPs). Mice were exposed to either a single or multiple doses of DEP by inhalation. We measured the mRNA level of the cytokines *Tnf *and interleukin-6 *(Il-6) *and the chemokines, monocyte chemoattractant protein *(Mcp-1)*, macrophage inflammatory protein-2 *(Mip-2) *and keratinocyte derived chemokine *(Kc) *in the lung tissue at different time points after exposure.

**Results:**

*Tnf *mRNA expression levels increased late after DEP-inhalation, whereas the expression levels of *Il-6*, *Mcp-1 *and *Kc *increased early. The expression of *Mip-2 *was independent of TNF if the dose was above a certain level. The expression levels of the cytokines *Kc*, *Mcp-1 *and *Il-6*, were increased in the absence of TNF.

**Conclusion:**

Our data demonstrate that *Tnf *is not important in early DEP induced inflammation and rather exerts negative influence on *Mcp-1 *and *Kc *mRNA levels. This suggests that other signalling pathways are important, a candidate being one involving *Mcp-1*.

## Background

When deposited in the lung, ultra-fine particles, i.e. particles with a diameter less than 100 nm, are probably more dangerous than large particles. The smaller the particles are, the greater surface area they have per mass unit: This implies that they have a greater chemical activity, can carry more PAH and other adsorbed chemicals, and they react more strongly with the lung tissue, thereby causing more inflammation than larger particles [1].

Several cytokines have been suggested to be involved in particle-induced inflammation. Focus has primarily been directed to the cytokines tumor necrosis factor (TNF) and macrophage inflammatory protein (MIP-2, also called CXCL2), which have been suggested to play an important role in particle-induced inflammation in the lung [2]. TNF itself has no chemotactic effect for neutrophils. Thus, the chemotactic effect of TNF is thought to be mediated through the induction of chemotactic cytokines, the so called chemokines. The chemokines, MIP-2 and keratinocyte derived chemokine (KC, also called CXCL1) are thought to be responsible for the recruitment of neutrophils in mice and exert their effect through binding to the same receptor CXCR2 [3]. MIP-2 and KC are considered to be murine homologues of the human interleukin-8 [4]. Furthermore, the chemokine macrophage chemotactic protein 1 (MCP-1, also called CCL2) has recently been suggested to be involved in DEP-induced inflammation [5]. MCP-1 acts by binding to the MCP-1 receptor (also called CCR2), which promotes both the maturation of monocytes to macrophages as well as their chemotactic recruitment [6]. Finally, the expression of interleukin-6 (*Il-6*) has been reported to be increased in particle-induced inflammation [7,8].

We recently reported from an inhalation study, that TNF was not required for DEP-induced inflammation when we evaluated the inflammatory effects after four repeated inhalation exposures in *Tnf -/- *and *Tnf +/+ *mice [7]. The aim of the present study was to examine the short term effects of DEP exposure, i.e. the effects up to two days after exposure. This was done 1) by evaluating the importance of *Tnf *and other inflammatory markers after exposure and 2) by investigating whether *Tnf *deficiency induces a change in the time course of inflammation induced by DEP. We evaluated the inflammatory response at the mRNA level, because cytokine expression seems to be regulated at the transcriptional level and by mRNA stability. Furthermore mRNA can conveniently be quantified in tissues. It has been shown that DEP up-regulates several genes involved in the inflammatory response, at both the mRNA and protein level in rats [5].

## Results

### Changes in cytokine expression over time

To evaluate the time course of particle-induced inflammation, we measured the expression of five cytokines (*Tnf*, *Il-6*, *Mip-2*, *Kc *and *Mcp-1*) in the lung tissue from BALBcJ (study 1) and C57xCBA mice (study 2) exposed to a single dose of DEP by inhalation at different time points. Study 1 consists of experiments with mice that we have reported of previously [8], and for which lung tissue in this study was reanalyzed for additional cytokines. Study 2 has not been reported of before. The results from the BALBcJ study and C57xCBA mice study are summarized in table 1 and table 2, respectively.

**Table 1:** Expression of cytokines in lung tissue from BALBcJ mice exposed to SRM1650

**Dose **	**Air**			**20 mg/m^3 ^DEP**			**80 mg/m^3 ^DEP**	
	
**Time**	**1 h**	**3 h**	**1 day**	**1 h**	**3 h**	**1 day**	**1 h**	**1 day**
*Tnf*	64 ± 23	41 ± 7.5	57 ± 43	49 ± 23	57 ± 7.7	100 ± 39	119 ± 70	178 ± 66**
*Il-6*	0.4 ± 0.3	0.2 ± 0.1	0.2 ± 0.1	1.0 ± 0.6**	0.2 ± 0.2	0.2 ± 0.01	3.5 ± 1.5**	0.9 ± 0.2**
*Mcp-1*	7.5 ± 4.7	21 ± 21	14 ± 20	8.5 ± 4.8	12.1 ± 3.4	11.6 ± 2.0	53.0 ± 57*	129 ± 86**
*Mip-2*	20 ± 10	12 ± 5.7	22 ± 17	27.5 ± 18	12.6 ± 1.2	32.2 ± 10.1	63.4 ± 17.4*	170 ± 66**
*Kc*	4.0 ± 1.5	3.6 ± 1.5	2.7 ± 1.4	16.0 ± 4.8**	4.5 ± 1.8	12.8 ± 6.5**	17.8 ± 2.1**	23.2 ± 11.5**

Expression of mRNA is normalized to 18S rRNA and is multiplied by 10^7^. Mean ± S.D. is shown (n = 3–7). IL-6 data has been published previously [8].

*P < 0.05 versus air-exposed mice.

**P < 0.01 versus air-exposed mice.

**Table 2:** Expression of cytokines in the lung tissue from C57xCBA mice exposed to 80 mg/m^3 ^SRM2975

**Exposure**	**Air**	**DEP**	
**Marker**		**1–6 h**	**1 day**

*Tnf*	49 ± 19	46 ± 14	149 ± 82**
*Il-6*	0.47 ± 0.25	5.6 ± 8.1**	2.1 ± 0.52**
*Mcp-1*	5.3 ± 1.9	18 ± 9.6**	16 ± 11*
*Mip-2*	23 ± 16	43 ± 37	105 ± 38**
*Kc*	3.5 ± 2.4	9.4 ± 6.7*	25 ± 18**

Expression of mRNA is normalized to 18S rRNA and is multiplied by 10^7^. Mean ± S.D. is shown (n = 6–11).

*P < 0.05 versus air-exposed mice.

**P < 0.01 versus air-exposed mice.

The *Tnf *response occurred relatively late. No change in *Tnf *mRNA levels was seen 1 hour after exposure to 80 mg/m^3 ^SRM1650 in BALBcJ mice compared to the mRNA level in mice exposed to filtered air, whereas the level was approximately 3-fold elevated (p < 0.01) 1 day later (Table 1). A marginal response was also only seen 1 day after the inhalation of 20 mg/m^3^. Similarly, in the experiment with the C57xCBA mice exposed to 80 mg/m^3 ^SRM2975 the expression level of *Tnf *was increased 1 day after exposure (3-fold, p < 0.01), whereas the expression level of *Tnf *was the same as in the air-exposed mice in the time-period from 1–6 hours after exposure (Table 2).

The expression level of the pro-inflammatory cytokine, *Il-6 *was elevated over the entire period in both the BALBcJ and C57xCBA mice after exposure to 80 mg/m^3 ^SRM1650 or SRM2975 (p < 0.01; Table 1 and 2). The highest level of expression was seen shortly after DEP inhalation (9-11-fold within six hours, p < 0.01) compared to the air-exposed, while the expression level was increased 4-fold (p < 0.01) 1 day after exposure to 80 mg/m^3^. Shortly after exposure 20 mg/m^3 ^SRM1650 *Il-6 *was temporarily elevated by 2.5-fold, showing a clear dose-response relationship (Table 1).

We measured the expression levels of the neutrophil chemotactic, chemokines, *Mip-2 *and *Kc *in BALBcJ mice exposed to a single dose of SRM1650 and C57xCBA mice exposed to a single dose of SRM2975. *Mip-2 *mRNA levels were increased in the BALBcJ mice exposed to 80 mg/m^3 ^both 1 hour (p < 0.05, 3-fold) and 1 day (p < 0.01, 8-fold) after exposure, whereas no change in expression levels was observed in the mice exposed to 20 mg/m^3 ^at any time point (Table 1). In the C57xCBA mice, the expression level of *Mip-2 *was only increased 1 day after inhalation (5-fold, p < 0.05) (Table 2). The expression level of *Kc *was statistically significantly increased in the BALBcJ mice exposed to 20 mg/m^3 ^1 hour as well as 1 day after exposure, while there was no increase 3 hours after exposure (Table 1). In both BALBcJ and C57xCBA mice exposed to 80 mg/m^3 ^the expression level of *Kc *was statistically significantly increased at both 1–6 hours and 1 day after exposure (Table 1 and 2). The expression level of the chemokine, *Mcp-1 *was increased approximately 3-fold in the C57xCBA mice 1 to 6 hours (p < 0.01) and 1 day (p < 0.05) after exposure (Table 2). The expression level of *Mcp-1 *was increased in the BALBcJ mice exposed to 80 mg/m^3 ^both 1 hour (7-fold) and 1 day (9-fold) after exposure (p < 0.01 and p < 0.05, respectively), whereas no increase in expression level was seen at any time point in mice exposed to 20 mg/m^3 ^(Table 1).

### TNF deficiency

In a second set of experiments, we investigated the specific effect of TNF. This was done by comparing the inflammatory response in *Tnf+/+ *and *Tnf-/- *mice after either a single dose of 20 mg/m^3 ^SRM2975 (study 3) or multiple doses of 20 mg/m^3 ^SRM2975 (study 4). Study 3 is a new animal experiment, whereas we have reported of the animal experiments in study 4 before [7], however, for which lung tissue was reanalyzed for new cytokines in this study.

### Single dose study in Tnf deficient mice

We studied the time course of inflammation by measuring the expression levels of the cytokines *Tnf*, *Il-6*, *Mip-2*, *Kc *and *Mcp-1 *in the lungs of the *Tnf+/+ *and *Tnf-/- *mice at 1 hour, 1 and 2 days after end of exposure to a single dose of 20 mg /m^3 ^SRM2975. In addition, we assessed the recruitment of inflammatory cells into the lung lumen. We chose to evaluate the time course after a single dose of 20 mg/m^3 ^SRM2975 in *Tnf+/+ *and *Tnf-/- *mice on the background of our previous results, that showed that *Il-6 *expression was significantly increased in BALB/CJ mice exposed to a single dose of 20 mg/m^3 ^SRM1650 [8]. The results from the single dose study using *Tnf *proficient and deficient mice are summarized in table 3.

**Table 3:** Inflammatory response in *Tnf+/+ *and *Tnf-/- *mice exposed to a single dose of 20 mg/m^3 ^SRM2975

**Marker**	**Exposure **	**Air**	**DEP**		
	
	**Mice**		**1 h**	**1 day**	**2 days**
*Tnf*	*Tnf +/+*	47 ± 15	40 ± 9.3	89 ± 36	74 ± 34
*Il-6*	*Tnf +/+*	1.1 ± 0.8	3.0 ± 1.9	1.3 ± 0.8	1.7 ± 0.7
	*Tnf -/-*	2.5 ± 1.6	4.8 ± 2.8	1.0 ± 0.4	2.6 ± 2.6
*Mcp-1*	*Tnf +/+*	9.4 ± 2.6	21.9 ± 9.2	9.2 ± 3.9	15.4 ± 3.9
	*Tnf -/-*	14.2 ± 9.1	39.5 ± 29.4	11.4 ± 4.4	33.5 ± 25
*Mip-2*	*Tnf +/+*	8.1 ± 0.8	11.9 ± 1.2	19.5 ± 8.2*	12.5 ± 2.6
	*Tnf -/-*	6.6 ± 2.5	10.0 ± 6.8	8.6 ± 2.2	10.4 ± 5.2
*Kc*	*Tnf +/+*	1.5 ± 0.5	1.9 ± 0.6	2.8 ± 0.8	2.3 ± 1.3
	*Tnf -/-*	1.3 ± 0.9	3.5 ± 2.2	1.5 ± 0.3	2.6 ± 0.7
Neutrophil (%)	*Tnf +/+*	0.2 ± 0.3	0.7 ± 0.3	3.8 ± 4.1**	7.3 ± 5.8**
	*Tnf -/-*	0.4 ± 0.8	1.1 ± 1.3	0.9 ± 0.8	1.5 ± 1.0

*Tnf+/+ *and *Tnf-/- *mice were exposed to a single dose of 20 mg/m^3 ^SRM2975.

Expression of mRNA is normalized to 18S rRNA and is multiplied by 10^7^. Mean ± S.D. is shown (n = 4–5).

*P < 0.05 versus air-exposed mice.

**P < 0.01 versus air-exposed mice.

In the *Tnf+/+ *mice the fraction of neutrophils in the BAL cells was strongly increased 1 (about 15-fold, p < 0.01) and 2 days after exposure (about 30-fold, p < 0.01) (Table 3). In the DEP exposed *Tnf-/- *mice there were 2-3-fold more neutrophils compared to the air exposed. This was not significantly different, but there was also no stastistical difference between knock out mice and wild type at any time point. We measured the expression levels of the cytokines *Tnf*, *Il-6*, *Mip-2*, *Kc *and *Mcp-1 *in the lung tissue. DEP exposure caused a 2-fold increase in *Tnf *mRNA levels in the *Tnf+/+ *mice 1 day after exposure compared to the air-exposed mice, although not statistically significant. As expected, no expression of *Tnf *was detected in the *Tnf-/- *mice. DEP exposure caused no significant change in expression levels of *Il-6*, *Mcp-1 *and *Kc *in neither the *Tnf+/+ *nor the *Tnf-/- *mice, at any time point. Although the difference was not statistically significant, the levels of *Mcp-1 *were higher in the *Tnf-/- *mice than the *Tnf+/+ *at the same time points. The level of *Mip-2 *expression in the DEP exposed *Tnf+/+ *mice was increased 1 day (p < 0.05) after exposure compared to the air exposed mice. The *Tnf-/- *mice did not respond to the DEP exposure with a higher *Mip-2 *expression.

### Repeated dose study in Tnf deficient mice

We studied the inflammatory response in *Tnf *deficient and proficient mice to repeated DEP doses by measuring the expression levels of *Mip-2*, *Kc *and *Mcp-1 *in the lung tissue from mice exposed to four bouts of inhalation of 20 mg/m^3 ^SRM2975 (Table 4). In both the *Tnf+/+ *and the *Tnf-/- *mice, DEP exposure increased the expression levels of *Mip-2 *around 3-fold (p < 0.5, p < 0.01). The expression level of *Kc *was significantly increased in the *Tnf -/- *mice (p < 0.01), but there was no increase of expression level in the *Tnf+/+ *mice. The *Mcp-1 *expression levels were increased 5-fold in the *Tnf *deficient mice (p < 0.01), and 1.5-fold in the *Tnf+/+ *mice although the latter was not statistically significant. In our recent publication [7], we analyzed the expression levels of *Tnf *and *Il-6 *mRNAs in the lung tissue and determined the fraction of neutrophils in the BAL cells. Results from this and the present study are summarized in table 4.

**Table 4:** Inflammatory response in *Tnf+/+ *and *Tnf-/- *mice exposed to multiple doses of 20 mg/m^3 ^SRM2975

**Marker**	**Exposure **	**Air**		**DEP**	
		
	**Mice**	***Tnf+/+***	***Tnf-/-***	***Tnf+/+***	***Tnf-/-***
*Tnf*		6.0 ± 4.4		13.8 ± 3.7*	
*Il-6*		0.6 ± 0.4	1.2 ± 0.6	1.5 ± 0.4*	4.2 ± 1.7**
*Mcp-1*		11.0 ± 6.1	7.5 ± 3.8	17.9 ± 10.3	33.8 ± 16.6**
*Mip-2*		6.5 ± 2.8	5.3 ± 2.5	22.9 ± 13.4**	14.9 ± 7.0**
*Kc*		2.1 ± 1.0	1.2 ± 0.7	2.5 ± 0.8	4.7 ± 2.0**
Neutrophil (%)		3.4 ± 6.7	0.2 ± 0.3	12.3 ± 7.9**	16.5 ± 10.1**

Expression of mRNA is normalized to 18S rRNA and is multiplied by 10^7^. Mean ± S.D. is shown (n = 7–8). Neutrophil, TNF and IL6 data has been published previously [7].

*P < 0.05 versus air-exposed mice. **P < 0.01 versus air-exposed mice.

## Discussion

It has been suggested that TNF is a key-player in particle-induced inflammation [2], and that TNF is an inflammatory mediator upstream of MIP-2 and KC [9]. However, in a previous study, we exposed *Tnf+/+ *mice and *Tnf-/- *mice to DEP by inhalation, and showed that TNF is not required for DEP-induced inflammation [7]. In the present study we provide further evidence that TNF is not important in early DEP-induced inflammation. Exposure to higher doses of DEP increased the expression level of *Mip-2*, independently of *Tnf *status, whereas enhanced *Kc *expression preceded *Mip-2 *expression only in *Tnf-/- *mice, which suggests that *Kc *may compensate for the deficiency of *Tnf*, or that TNF exerts negative feedback on KC signalling. The DEP-induced expression of *Mcp-1 *and *Il-6 *occurred in the absence of *Tnf*. Our results indicate that other signalling pathways, perhaps involving MCP-1, are important for early DEP induced neutrophilic inflammation.

Thus, we evaluated other signalling patways with the expression of four additional cytokines, *Il-6*, *Mip-2*, *Kc *and *Mcp-1*, in the early and late phase of the DEP-induced inflammatory process. The results from the BALBcJ [8] and the C57xCBA mice studies, show very clearly that *Il-6 *is expressed early in inflammation (1–6 hours after exposure) and that the expression of *Il-6 *has declined a day after exposure, although it is also elevated after repeated exposures.

The chemokines *Mip-2 *and *Kc *are strongly coupled to the influx of neutrophils in the rodent lung [4], whereas MCP-1 is chemotactic for monocytes. MIP-2 and MCP-1 have both been linked to the inflammatory process in response to particles [2,5]. All of these three cytokines were more expressed 1 hour after exposure to a high dose (80 mg/m^3^) independently of mouse strain or type of DEP. *Mip-2 *levels increased further 1 day after exposure and this also included the low dose of SRM2975, whereas a further increase in *Mcp-1 *mRNA levels only occurred after SRM1650 in BALBcJ mice and not with SRM2975. The mRNA levels of *Kc *were maximal at 1 hour after exposure to SRM1650 and even showed a decrease 3 hours after exposure to 20 mg/m^3^, although there was a slight further increase 1 day after exposure to 80 mg/m^3 ^SRM2974. Only the *Kc *level was significantly increased 1 hour after the low dose of 20 mg/m^3 ^SRM1650 in BALBcJ mice. Accordingly, *Il-6*, *Mip-2*, *Mcp-1 *and *Kc *are cytokines induced in the early phase of DEP-induced inflammation, while *Tnf *is expressed late at about a day after exposure. *Mip-2 *may have a more important role in the later phase as judged by the further increased expression 1 day after exposure. The results may be consistent with that DEP activates Toll like receptor 4 (TLR4) signalling because the *Tnf *response occurs late in this pathway. In fact, there are indications that air pollution particles stimulate the TLR4 pathway [10-12]. Bacterial lipopolysaccharide (LPS), the best studied stimulator of the TLR4 pathway, binds to the TLR4 and activates nuclear factor κB (NF-κB) mediated neutrophil inflammation. The TLR4 signals in two parallel pathways, both leading to activation of NF-κB. The MyD88 adaptor dependent pathway directly activates NF-κB, whereas in a later pathway, dependent on interferon-β adaptor molecule and probably on interferon-regulatory factor 3, NF-κB activation is dependent on *de novo *synthesis, secretion, and autocrine signalling by TNF over the TNF receptors [13,14]. Moreover, a few studies support our results that TNF is of no or minor importance in particle-induced inflammation in the lungs of rodents. E.g., recently, a highly increased expression levels of the chemokines, *Mcp-1 *and *Mip-2*, but no change in *Tnf*, was found in BAL cells isolated from DEP (SRM2975) exposed rats [5]. This is in accordance with, that BALF from rats exposed to quartz by intratracheal instillation contained increased MIP-2 protein levels over the measured time period from 3 to 90 days after exposure, whereas TNF protein levels remained under the detection limit [15]. However, when comparing our study with the above mentioned studies, it should be noticed that they describe the long term effects of particles, i.e. the effects in a time window from 1–30 and 3–90 days after exposure, respectively, while this study describes short term effects of particles, i.e. 1 hr to 2 days after exposure.

As we have previously reported, *Il-6 *was upregulated upon repeated DEP exposure both in *Tnf+/+ *and *Tnf-/- *mice [7]. This might be consistent with a report showing that SRM2975 increased IL-6 in BALF, whereas no change in TNF protein levels were observed [16]. Although not statistically significant, the *Il-6 *expression level was higher 1 hour after exposure in both *Tnf+/+ *mice and *Tnf-/- *mice after a single dose exposure.

In the repeated dose TNF study, both the *Tnf+/+ *and *Tnf-/- *mice responded with an increase in *Mip-2 *response in the lungs. The *Mip-2 *induction was similar in *Tnf+/+ *and *Tnf-/- *mice. This is surprising because MIP-2 was believed to be an inflammatory marker downstream of TNF [9]. MIP-2 has been strongly linked to the influx of neutrophils [2]. In the single dose TNF study, the level of expression of *Mip-2 *was increased in the *Tnf+/+ *mice 1 day after exposure and borderline significant 2 days after exposure. The *Mip-2 *expression tended to correlate with the influx of neutrophils, because the influx of neutrophils was statistically significant 1 day and 2 days after exposure. Similarly, TNF receptor 1 deficient mice had an intact neutrophil infiltration after pulmonary exposure to silica [17]. No increase in *Mip-2 *mRNA levels was seen in the single dose exposed *Tnf -/- *mice at any time: Possibly, there is a threshold for the induction of *Mip-2 *in the *Tnf -/- *mice, because the repeated exposure in the *Tnf *deficient mice resulted in a *Mip-2 *response. In the repeated dose experiment, the expression of *Kc *was only increased in the *Tnf -/- *mice, which together with the results from the BALBcJ and C57xCBA mice suggests that TNF exerts an inhibitory effect on *Kc *expression.

MIP-2 and KC are both chemotactic for neutrophils and have been suggested to be redundant [18]. MIP-2 has a higher affinity to the CXCR2 receptor than cytokine-induced neutrophil chemoattractant (CINC), the rat analogue to KC, and is a more potent chemotactic factor for neutrophils [19]. The expression of *Kc *increased earlier than *Mip-2*. This is consistent with a recent report that CINC (KC) apparently functions as a priming signal to the neutrophils, which afterwards are attracted to the more potent but local chemoattractant MIP-2 in the lung [20]. Our results show that both *Mip-2 *and *Kc *are increased in the absence of *Tnf*. Both *Mcp-1 *and *Kc *mRNA levels were induced to lesser extent by DEP in the *Tnf+/+ *than in the *Tnf-/- *mice. This might indicate TNF exerts negative feedback on these cytokines.

Recently, it was reported that primary embryonic fibroblasts from *Tnf-/- *mice had intact LPS induction of *Mcp-1 *expression levels [13]. Knock out mice with abrogated MCP-1 signalling have indeed impaired macrophage and neutrophil infiltration upon some toxic stimuli to the lung [9-11]. This calls for further research into the importance of TLR4, MCP-1 and other early response signalling in particle-induced inflammation.

In one of our experiments we used the SRM1650 material which is particulate matter collected from a heavy-duty diesel engine. Because this is not available any more, we used SRM2975 in the rest. SRM2975 is a DEP preparation collected from a diesel-powered industrial forklift. The DEP materials differ in their chemical composition. E.g., SRM 1650 contain a higher amount of metals and PAHs than SRM 2975 [21,22]. Although we have not compared this in depth, similar responses were obtained using the two different DEP preparations although the SRM1650 seemed to give a stronger response particularly with respect to MCP-1.

These experiments were performed using several different mouse strains. There were minor differences in expression patterns, which may be ascribed to strain or DEP material effects. The overall picture was consistent across mouse strains, indicating that the observed effects are not strain specific.

## Conclusion

In summary, we have shown that TNF is not important in early DEP-induced inflammation. Exposure to DEP by inhalation caused an increased expression level of *Mip-2*, that was independent of *Tnf *status, if the dose was above a certain level. *Kc *expression seemed to precede the *Mip-2 *expression and *Kc *was only expressed at a higher level in the DEP-exposed *Tnf-/- *mice, which suggests that KC may compensate for the deficiency of TNF, or that TNF exerts negative feedback on KC signalling. The DEP-induced expression of *Mcp-1 *and *Il-6 *was increased in the absence of *Tnf*. Our results indicate that TNF signalling is not important for early DEP induced neutrophilic inflammation. Attention should be focused on other pathways, a possible candidate being one involving MCP-1.

## Methods

### Animals

*Tnf-/- *mice (B6, 129S-Tnftm1Gk1) were obtained from JAX Mice (USA). C57 BL/6J mice were used as *Tnf+/+ *mice. C57 BL/6J and BALBcJ mice were purchased from Taconic Europe, Denmark. C57xCBA mice that express luciferase under the control of NFκB [23] were obtained from Rune Blomhoff, Norway (hereafter referred to as C57xCBA mice). The mice were allowed to acclimatize for 2 weeks and were given food (Altromin 1324) and water ad libitum. The mice were group housed in polypropylene cages with sawdust bedding at controlled temperature 21 ± 1°C and humidity 50 ± 10% with a 12-h light:12-h dark cycle. Female *Tnf-/- *and *Tnf+/+ *mice were studied at 9–11 weeks of age. Both male and female C57xCBA mice were studied at 7–8 weeks of age. Female BALBcJ mice were studied at 8 weeks of age.

### Particles

DEP were Standard Reference Material 2975 and 1650 from the National Institute of Standards and Technology (NIST)(Gaithersburg, MD, USA). SRM1650 are combustion particles from a heavy duty diesel engine. SRM2975 are DEP collected from a diesel-powered industrial forklift. A detailed description of the particles can be viewed at [22].

### Exposure of mice

The study consists of four parts: 1) a single exposure of BALBcJ mice to 20 or 80 mg/m^3 ^SRM1650, 2) a single dose exposure of C57xCBA mice to 80 mg/m^3 ^SRM2975, 3) a single dose exposure of *Tnf-/- *mice and *Tnf+/+ *mice to 20 mg/m^3 ^SRM2975, and 4) four repeated exposures of *Tnf*-/- mice and *Tnf*+/+ mice to 20 mg/m^3 ^SRM2975. In all four parts, the mice were exposed by inhalation for 90 minutes either one single time (study 1, 2 and 3) or repeated on each of four consecutive days (study 4) in an 18 l nose-only exposure chamber.

1) The design of the single dose exposure of BALBcJ mice has been described elsewhere [8]. In summary, the mice were exposed to either 20 mg/m^3 ^or 80 mg/m^3 ^SRM1650 and the endpoints were analyzed 1 hour, 3 hours or 1 day (22 hours) after exposure.

2) C57xCBA mice were exposed to a single dose of 80 mg/m^3 ^SRM2975. Endpoints were analyzed at different time points.

3) In the third study, the mice were exposed a single time to 20 mg/m^3 ^SRM 2975 at similar conditions as the repeated exposure. Endpoints were analyzed 1 hour, 1 and 2 days after end of last exposure. Study 2 and our previous studies show that the levels of expression of inflammatory cytokines are unchanged over time in the air-exposed mice [8]. On that background and because of ethical considerations, we chose only to evaluate the air-exposed mice at a single time point.

4) The design of the repeated exposure has been described in detail elsewhere [7]. In brief, we exposed *Tnf-/- *mice and *Tnf+/+ *mice by inhalation to 20 mg/m^3 ^DEP or filtered air for 90 min on each of four consecutive days. One hour after the last inhalation the mice were killed and organs were snap frozen in liquid nitrogen and stored at -80°C. Endpoints were analyzed.

The particles were aerosolized by a microfeeder with dispersion nozzle (Fraunhofer Institut für Toxikologie and Aerosolforschung, Hannover, Germany). The number of particles was measured by a condensation particle counter (TSI model 3022A). The numbers of particles in the single dose TNF study and C57xCBA study were about 9.1·10^5^/cm^3 ^and 3.0·10^6^/cm^3^, respectively. Particle numbers in the multiple-dose TNF study and the BALBcJ study are described elsewhere [7,8]. The particle concentration was measured four times during exposure by weighing of filters. The particle concentrations in the single dose TNF study and C57xCBA study were approximately 20 mg/m^3 ^and 80 mg/m^3^, respectively.

After exposure, tissue and BAL cells from the mice were for all studies prepared as described previously [7,8].

### Preparation of RNA and cDNA from lung tissue

RNA from the multiple dose experiment was prepared using NucleoSpin 96 RNA kit (Macherey-Nagel) as described previously [7]. RNA from the three single dose experiments were prepared using QIAamp^® ^RNA Blood Mini Kit (Qiagen, USA) as described by the manufacturer. cDNA was prepared from DNase treated RNA using TaqMan reverse transcription reagents (Applied Biosystems, USA) as described by manufacturer.

### Real time RT-PCR

The *Tnf*, *Mip-2*, *Kc*, *Il-6 *and *Mcp-1 *gene expression was determined using real-time RT-PCR with 18S RNA as the reference gene. Each sample was run in triplicate on the ABI PRISM 7700 sequence detector (PE Biosystems, Foster City, CA, USA). For TNF, IL6 and MCP-1, TaqMan pre-developed reaction kits (Applied Biosystems, USA) were used. Unfortunately, the TaqMan pre-developed reaction kit for determination of TNF, we previously used [7], is not commercially available anymore. Therefore we had to change to another TNF kit: nr 4331182 (Applied Biosystems, USA). This means that is not possible to compare the results of TNF in this publication with the results published in our former publication [7]. The expression levels of *Tnf *are approximately 5-fold higher when using the new kit. For MIP-2 and KC, probes and primers were designed as described previously [24]. However, the KC sense primer was modified at two nucleotide positions to: 5'-gtg tct agt tgg tag ggc ata at-3', because the assay did not work with the published primer [24]. This was probably due to interspecies variations. In all assays TaqMan pre-developed mastermix (Applied Biosystems) was used. For the KC-assay the MgCl_2 _concentration was adjusted to 3.0 mM. Target and 18S RNA levels were quantified in separate tubes. The target genes were normalized (ΔC_t_) to 18S RNA by subtracting the cycle threshold value of 18S RNA (C_treference_) from the Ct value of the gene of interest (C_ttarget_), i.e. ΔC_t _= C_ttarget _- C_treference_. The relative expression of the target gene was calculated by the comparative method 2^-ΔCt^[25]. The average standard deviation on triplicates was 15 %. The standard deviation on repeated measurements of the same sample (the control) in separate experiments was 20 %, indicating that the day-to day variation of the assay was 20 %. The probes and primers have been validated and the PCR was shown to be quantitative over a range of 32 or 64-fold range. Messenger RNA measurements were excluded if the 18S content fell outside the range in which the PCR was found to be quantitative defined by the validation experiments. Negative controls, where RNA was not converted to cDNA, were included in each run.

### Statistical analysis

Data from the study 1, 3 and 4 were analyzed by two-way analysis of variance. Data from study 2 were analyzed by one-way analysis of variance. To fulfil the criteria for normality and variance homogeneity some of the markers were logarithmically transformed once or twice. For some markers, the data were ranked before a nonparametric test, because it was not possible to transform the data in a way that satisfied the criteria for normality and variance homogeneity. To determine the statistical significance of biomarkers, pairs of interest were compared with Tukey's Studentized range test with control of type 1 experimental error. In study 3, one mouse was excluded as an outlier because the data points for this mouse were more than 2 standard deviations from the rest of the data for all endpoints except one. In study 2 the air-exposed mice were grouped. Statistical analysis was performed with the SAS 8.2 statistical program.

## Competing interests

The author(s) declare that they have no competing interests.

## Authors' contributions

ATS carried out the real-time RT analysis, performed the statistical analysis and drafted the manuscript. NRJ, JBO, SLK, LR and MD carried out the exposure of the mice. ATS, SL, UBV and HWA conceived of the study and participated in its design and coordination. HWA, UBV and SL helped to draft the manuscript. All authors read and approved the final manuscript.
